# Characterizing internet health information seeking strategies by socioeconomic status: a mixed methods approach

**DOI:** 10.1186/s12911-016-0344-x

**Published:** 2016-08-09

**Authors:** Susan L. Perez, Richard L. Kravitz, Robert A. Bell, Man Shan Chan, Debora A. Paterniti

**Affiliations:** 1 School of Nursing Science and Health Care Leadership, University of California, Davis, USA; 2 Department of Internal Medicine, University of California, Davis, USA; 3 Department of Communication, University of California, Davis, USA; 4 Department of Sociology, University of California, Davis, USA; 5 Department of Public Health Sciences, University of California, Davis, USA; 6 Department of Kinesiology and Health Science, California State University, Sacramento, 6000 J Street; Solano Hall 2003, Sacramento, CA 95819-6073 USA; 7 Department of Sociology, California State University, Sonoma, USA

**Keywords:** Internet, Heuristics, Health information seeking

## Abstract

**Background:**

The Internet is valuable for those with limited access to health care services because of its low cost and wealth of information. Our objectives were to investigate how the Internet is used to obtain health-related information and how individuals with differing socioeconomic resources navigate it when presented with a health decision.

**Methods:**

Study participants were recruited from public settings and social service agencies. Participants listened to one of two clinical scenarios – consistent with influenza or bacterial meningitis – and then conducted an Internet search. Screen-capture video software captured the Internet search. Participant Internet search strategies were analyzed and coded for pre- and post-Internet search guess at diagnosis and information seeking patterns. Individuals who did not have a college degree *and* were recruited from locations offering social services were categorized as “lower socioeconomic status” (SES); the remainder was categorized as “higher SES.” Participants were 78 Internet health information seekers, ranging from 21–35 years of age, who experienced barriers to accessing health care services.

**Results:**

Lower-SES individuals were more likely to use an intuitive, rather than deliberative, approach to Internet health information seeking. Lower- and higher-SES participants did not differ in the tendency to make diagnostic guesses based on Internet searches. Lower-SES participants were more likely than their higher-SES counterparts to narrow the scope of their search.

**Conclusions:**

Our findings suggest that individuals with different levels of socioeconomic status vary in the heuristics and search patterns they rely upon to direct their searches. The influence and use of credible information in the process of making a decision is associated with education and prior experiences with healthcare services. Those with limited resources may be disadvantaged when turning to the Internet to make a health decision.

## Background

Despite implementation of the United States’ Affordable Care Act to expand access to healthcare services, barriers to healthcare still include finding continuity of care (e.g., a consistent primary care provider); accessing care in a timely manner (e.g., ability to get an appointment in a timely manner); gaining access to a site of care where needed services are readily available (e.g., transportation); and inability to pay for services (e.g., lack of insurance) [1, 2]. These and other persistent barriers can leave individuals with unanswered questions about health matters. Finding answers to such questions may necessitate a broad range of health-seeking strategies including attention to resources outside the formal healthcare system.

Improved access to care has the potential to expand choices in care, [3] yet the ability to capitalize on expanded choices for decision making is only as promising as an individual’s ability to access timely, accurate information that is relevant to the person’s situation. Those with socio-economic resources, health literacy, and experience with the healthcare system have a greater capacity for navigating health information sources to inform decision making that promotes their health than those who do not [4]. Little is known about the influence of Internet health information during the process of making health decisions, such as the process for deciding whether to seek-out health care services [5].

When confronted with troubling symptoms, people who have limited access to health care services due to lower SES must weigh the severity of their symptoms in the context of healthcare access barriers. While the Internet is not intended to replace traditional care, the Internet might be one of few available sources of information for those with limited access to healthcare services. The Internet is a prime source for obtaining health-related information, [6, 7] such as providing options on how to access health care services and health care professionals [8]. Online information is especially valuable for those with limited resources because its low cost and wealth of information [7]. For those lacking resources, the Internet could be a powerful tool for interpreting symptoms and guiding decision making about care. Still, little is known about how individuals make use of the Internet to inform decisions related to health-related care and care access [5].

Paradigms in psychology of judgment and decision-making may help describe how people seek information in response to a health concern [9]. One such paradigm is dual-processing theory, which posits there are two approaches to processing information—intuitive and deliberative [10]. Those who use intuitive processing are likely to activate a number of potential biases and heuristics, while those who process information using a deliberative approach are more methodical in their evaluation of information presented [10].

Despite its attractions, critics have warned that online health information is highly variable in readability, completeness, and accuracy [11]. We investigated how the Internet is used to obtain health-related information and how individuals of different socioeconomic status navigate it. To examine individuals’ Internet searching processes, we created two clinical vignettes, each portraying an acute illness of different clinical severity. These vignettes were presented to research participants with differing levels of socioeconomic status defined by education and use of social services. Participants searched for information based on these scenarios using the Internet as a resource. We then qualitatively identified the information-seeking components that influenced health-related information seeking based on an individual’s socioeconomic status.

## Methods

### Recruitment of participants

Participants were recruited and data collected between March and August 2013. Potential participants were identified at locations offering social services (e.g., soliciting door-to-door in a low-income housing community, social services offices, and community resource fairs) and locations not offering social services (e.g., University listservs, student/family housing, and flyers in local coffee shops). All individuals interested in participating completed a brief, online screening questionnaire that included basic demographic questions, including level of education and insurance status, as well as health care access questions [2]. A detailed description of the online screening questionnaire has been provided elsewhere [12].

Eligible participants were 21 to 35 years of age, had searched the Internet for health information within the past 12 months, and reported at least one barrier to health care services access, including inability to get an appointment in a timely manner, challenges with transportation to see a health care provider, no consistent primary care provider, or inability to pay for services [2]. Study participants received $20 compensation for their time.

### Data collection

Participants were randomly assigned to one of two clinical symptom scenarios to prompt their Internet search: (1) fever, mild headache, dry cough, and myalgia (suggestive of influenza); or (2) fever, severe headache, and stiff neck (suggestive of meningitis). Symptom scenarios were developed based on Centers for Disease Control (CDC) guidelines and input from the clinical co-author (RLK) and study consultants. Both symptom scenarios were pilot-tested for face validity and understanding in a small sample of adults (*n* = 8) who fit inclusion criteria for study participation. Following randomization to one of the two scenarios, the lead author (SLP) asked participants, “What do you think you are experiencing?” – for judgment about etiology – and then instructed them to “Search the Internet, as though [you were] experiencing this situation.” The lead author also trained all participants how to narrate their processes of searching and decision-making. All study participants were instructed to “think aloud” while conducting their search for information related to the clinical scenario. At the end of their search, participants were again asked to identify their perceived symptom etiology (which was stated in terms of their response to the question “What do you think you are experiencing?”).

Participants had a choice of web browser to conduct their search: Firefox, Internet Explorer, or Google Chrome. Web browsers opened to a blank page. Internet searches and participants’ “think-aloud” vocalizations were digitally recorded using screen capture video-recording software [13], which also captured mouse clicks and keystrokes. Between each search session, web browser search history and cookies were deleted. Audio recordings were transcribed verbatim for content analysis.

### Coding of internet search behaviors

Search-related mouse clicks and keystrokes making up the step-by-step process of Internet searching and decision-making processes were examined and coded as unique components of the search process. Each mouse click and combination of keystrokes (Internet searching) was coded as related to assessing one of the following “search pattern components”: cause of the symptoms (etiological assessment), defining the meaning of symptoms (symptom exploration), or characterizing a pattern of action based on the symptoms (treatment seeking). Etiological assessment describes testing a diagnostic hypothesis (i.e., entering the search term “meningitis” or clicking on a hyperlink titled “Flu”). Symptom exploration describes searching that involves using symptoms to guide the search (e.g., “achy, high temperatire [sic], sore muscles” or clicking on a link “cough, muscle pain”). Treatment seeking describes searching for remedies, recommended actions or alerts such as recommendations for seeking immediate care from a health care provider, looking for a cure, or searching for health care services (i.e., entering the search term “flu remedies” or selecting the link “when to seek Medical Care”). These coding patterns were grouped into broad thematic categories for analysis (characterizing search-related motivation and decision making) and analyzed for the number of times participants switched (switching) between search pattern components. Perez et al. (2015) provide examples and detailed explanation of this methodology.

Searches that consisted of entering at least two different search terms and then clicking or selecting at least one link beyond one search were labeled ‘branching.’ Searches that consisted of entering less than two searches and did not select a link beyond a search were labeled as ‘pruning.’

### Analyses of participants and search strategies for understanding symptoms

Study participants who had no college degree *and* who were recruited from sites offering public services (e.g., employment, housing, welfare services, and food stamps) were identified as lower socioeconomic status (lower-SES); those with a Bachelor’s degree or any post-graduate educational experience, regardless of recruitment site, were classified as higher socioeconomic status (higher-SES). Education is a reflection of a range of noneconomic social characteristics, such as general and health-related knowledge, literacy, and problem-solving skills, with important health effects [14]. In addition to education, recruitment site was used as a proxy for resource utilization, access, and dependency.

Participants’ etiological assessments consisted of their “best guess at the diagnosis.” Assessments were coded as “correct,” if the “best guess” matched the diagnosis of the assigned scenario; “incorrect,” if the “best guess” did not match the assigned scenario; and “unsure,” if a study participant stated that they “didn’t know,” were “confused,” or “unsure” about what they were experiencing.

Next, we classified the differences between pre- and post-search etiological assessments. Etiological assessments that did not change as a result of the Internet search were coded as “no influence”; for etiological assessments where there was a change in decision from the initial decision (i.e., from correct to incorrect, incorrect to correct, unsure to either correct or incorrect, or correct/incorrect to unsure) were coded as “any influence.”

Finally, we examined the relationship between individual socioeconomic status (lower-SES versus higher-SES) and influence of the Internet search on decision (“no influence” vs. “any influence”) using Pearson’s *χ*^2^ test of significance. Building on previous research that concluded that there are two types of Internet search behaviors for processing information – *intuitive* (i.e., unconscious, rapid, automatic, and high capacity thin) and *deliberative* (i.e., conscious, slow, and deliberative processing) [12], we examined the relationship between socioeconomic status (lower-SES versus higher-SES) and information processing (intuitive vs. deliberative), again using Pearson’s *χ*^2^ test of significance.

All statistical analyses were performed using SAS(r) software version 9.3 (SAS Institute, Cary, NC).

### Qualitative analysis of “think aloud” processes

“Think aloud” transcripts for the 78 study participants were systematically reviewed for important information searching components by two of the authors (SLP and DAP) using iterative content analysis. First, transcripts were examined for participant expression of perceived etiology. Next, “think aloud” data were assessed for the following: a) use of search terms, b) selection of websites, c) articulation of rationale for information selection or search strategy, d) perception of website credibility, e) mention of previous experiences and knowledge, f) attention to information formatting, including illustrations, g) articulation of frustration and confusion, and h) assessment of symptoms during search. Coded information was grouped into discrete categories, and coherent themes and patterns that described decision-making processes. Authors DAP and SLP then reviewed thematic content and patterns and agreed on a final categorization of themes that reflected Internet decision-making processes and search-related motivation.

## Results

Of the 124 individuals who contacted the research team for potential participation in the study, 99 (80 %) were study eligible. Seventy-eight of the 99 eligible persons (79 %) completed all parts of the study, including the Internet search and “think aloud” interview. The final sample size was thus *N* = 78.

### Participant demographics and characteristics

Table 1 presents demographic characteristics for study participants, as compared with the county residents where participants were recruited. Twenty-six (33 %) of the participants were classified as lower-SES because they did not have a bachelor’s degree *and* were recruited from sites offering social services (e.g., employment, housing, welfare services, and food stamps). The remaining 52 participants (67 %) were identified as higher-SES; 13 of these individuals (17 %) had a bachelor’s degree and were recruited from sites offering social services, 26 (33 %) did not have a bachelor’s degree and were recruited from sites that did not offer social services (e.g., University campus), and 13 (17 %) had a bachelor’s degree and were recruited from sites not offering social services.

**Table 1:** Sample demographic characteristics (*N* = 78)

Category	Study Participants	Yolo County	*p*-value
Mean Age (in years)	25	30^c^	<0.01
Gender			<0.01
Male	29 % (23)	49%^a, d^	
Female	71 % (55)	51%^a, d^	
Race			<0.01
White	29 % (23)	76%^a^	
Other	71 % (55)	24%^a^	
Ethnicity			<0.01
Hispanic	47 % (37)	31%^a^	
Other	53 % (41)	69%^a^	
Education			0.40
No Bachelor’s Degree	66 % (52)	62%^a^	
Bachelor’s Degree or higher	35 % (26)	38%^a^	
Insurance status			0.02
Uninsured	23 % (18)	13 % ^b^	
Public Insurance	14 % (11)	19 % ^b^	
Other Insurance	63 % (49)	68 % ^b^	

^a^2010 US Census

^b^Among individuals under the age of 65 taken from the 2005 California [23]

^c^Yolo county data taken from City-data.com [24]

^d^Data is specific to those between the ages of 20 and 34 years [23]

### Type of search strategy

The average search length was 308 s. Those identified as lower-SES took longer (*M* = 326 s, *SD* = 68) to complete the Internet search task than those identified as higher-SES (*M* = 299 s, *SD* = 138) (*p* < .000).

**Fig. 1:**
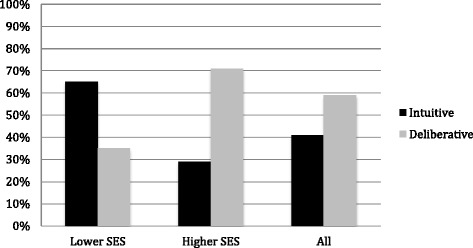
Information processing strategies based on Internet searching by lower-SES and higher-SES. We examined the relationship between socioeconomic status and type of information processing strategy (intuitive vs. deliberative). Those who use intuitive processing are likely to activate a number of potential biases and heuristics, while those who process information using a deliberative approach are more methodical in their evaluation of information presented

We classified 41 % of participants as employing an intuitive approach and 59 % a deliberative approach. We examined the relationship between socioeconomic status and type of information processing strategy (intuitive vs. deliberative). Compared with their higher-SES counterparts, lower-SES participants were much less likely to employ deliberative processing (35 % versus 71 %, *p* = .002) (Fig. 1). As a result of their varying strategies, higher-SES individuals averaged 2.52 switches (*SD* = 2.60) between search pattern components (e.g., etiological assessment, symptom exploration, and treatment seeking) than their lower-SES counterparts (*M* = 1.23 switches, *SD* = 1.77, t(76) = 2.27, *p* = .03).

### Differences in pre- post-assessment of symptom etiology

Next, we examined whether Internet searching influenced a change in participants’ judgments of symptom etiology. In response to being asked to guess the etiology of the symptoms presented in the vignette, approximately one-third of participants (35 % of the higher-SES group and 27 % of the lower-SES group) guessed the symptom etiology correctly. “Correct”guesses were characterized as “choosing the appropriate cause of the symptoms presented in the vignette” after a single or a series of guesses. Among those initially guessing correctly, 2 higher-SES participants and 1 lower-SES participant changed their guess to “incorrect” following their internet search. Among those initially guessing incorrectly, 7 higher-SES participants and 3 lower-SES participants changed their guess to “correct.”

**Fig. 2:**
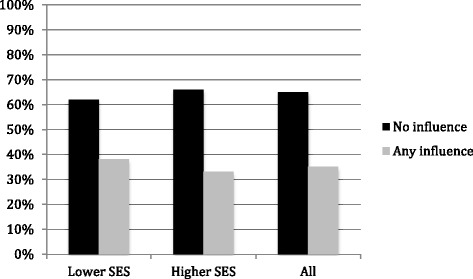
Influence on decision-making about symptom scenarios based on Internet searching by higher-SES and lower-SES. Etiological assessments that did not change as a result of the Internet search were coded as “no influence”; for etiological assessments where there was a change in decision from the initial decision (i.e., from correct to incorrect, incorrect to correct, unsure to either correct or incorrect, or correct/incorrect to unsure) were coded as “any influence.” When comparing the higher-SES with lower-SES, there was no significant difference for whether a participant changed their mind about the cause of their symptom etiology as a result of Internet searching (*p* = .62)

Among all of the participants, 51 participants (65 %) were not influenced by their Internet search for health-related information (no influence) (Fig. 2). When comparing the higher-SES with lower-SES, there was no significant difference for whether a participant changed their mind about the cause of their symptom etiology as a result of Internet searching (*p* = .62).

### “Think aloud” processing key findings

Systematic iterative review of digital recordings and transcripts for “think aloud” interviews uncovered three search strategy decision-making heuristics used in Internet health information searching. The three heuristics were: a) prior clinical or symptom-related experience, b) credibility of search-related information, and c) Internet story coherence. Table 2 defines and illustrates each type of heuristic.

**Table 2:** Heuristics of decision-making related to Internet searching

Heuristics	Definition	Example from “think aloud” interview
Prior clinical or symptom related experience	previous interactions with a health care provider, prior experiences with symptoms (self or familiar other)	“I would—this would probably be where I would reflect on what my personal experience would have caused me to feel this way, and I would—if I started feeling really terrible”
		“Then I’ll search for symptoms of the flu, because I didn’t get my flu shot, so I have to look into that.”
		“I want to check silent migraines, sometimes I get those, and see what happens and why it triggers it.”
Credibility of information source	recognition of information status or contributors to the website, organization or format of the website, and resonance with participant	“We're going to go to CDC for seasonal influenza because I feel like that would be a beneficial type of information.”
		“I’m going to use Wikipedia, even though it’s frowned upon.”
		“I’d probably click on flu and oh, the first one says flu.gov. So, a government site might have some accurate information.”
Internet story coherence	consistency in information presented with own biases, vignette symptom definition, prior history with symptoms, or information obtained during search	“This is sounding a little bit closer to what I’m experiencing.”
		“And this is pretty much where I would probably stop because it says right here the common cold, bronchitis or a viral syndrome.”
		“There’s a lot of the same or the similarities between the flu and my symptoms, so I’m going to keep that on the table and think that maybe I have the flu, but go look at some other diseases.”

All participants had some *prior clinical or symptom experience* by which they assessed the relevance of, and made decisions about, their Internet search. Participants relied on *prior experiences* with symptoms or illness where they discussed a course of action or treatment with a health care provider and/or trusted friends or family.

Participants also determined the perceived *credibility of information source* to guide their Internet search processes. The *credibility of information source* heuristic relied on participants’ assessment of three aspects: a) status of the site or contributors to the site, b) site design, and c) familiarity or resonance with the participant. *Status of the site or contributors* meant recognition of website address features, such as the Internet’s top-level domains, including “.gov,” “.edu,” “.org” domains, or website hosts, such as CDC or Wikipedia, to assess credibility of a website. *Site design* involved the aesthetic appeal or ease with which the participant could engage with the website, including site organization, layout, and “professional” display. *Familiarity or resonance with the participant* included any information or informational quality that made the information personally meaningful to the participant, including a previous experience with the website or information presented on the website. Table 3 defines and further illustrates each type of *credibility of information source* heuristic from participant “think aloud” sessions. As participants search the Internet and encounter various pieces and types of information, they are confronted with having to continually assess whether the information presented to them makes sense in the context of their prior experience and source credibility *and also* creates a coherent story as each piece of information is added or ignored. The third heuristic, *Internet story coherence*, characterizes the participants’ process of sense making during their Internet search. Using this heuristic, the participant draws on Internet information as it arises to validate a course of decision-making so that, in the end, the final decision might be explained rationally.

**Table 3:** Credibility aspects and examples of these aspects

Aspects of Credibility	Justifications from “think aloud” sessions
*Status of the site or contributors*	“I’m going to go to the Mayo Clinic website on the common cold because Mayo Clinic seems like a really kind of trustworthy place.
	“I’d probably click on flu and, oh, the first one says flu.gov. So, a government site might have some accurate information.”
*Site design*	“So usually, maybe I’ll look and see the first couple sites that come up if something sticks out to me as looking more professional, I would go with that.”
	“This kind of has cute little pictures and stuff and it shows a person with the stomach flu like they have stomach cramps.”
*Familiarity or resonance with the participant*	“Heard of this website.”
	“Familydoctor.org, health forum, meningitis. That sounds like that rings a bell.”

### Internet search strategies and organization of heuristics

Although no significant differences existed between the lower-SES and higher-SES in the influence of their Internet search related to symptom etiology, the lower-SES and higher-SES participants engaged in different types of information processing during Internet searches. Across participants, the higher-SES were more likely than their lower-SES counterparts (31 % versus 60 %, *X*^*2*^ (1, N = 78) = 5.77, *p* < .05) to engage in branching searches.

Table 4 qualitatively characterizes for two study participants the branching (Participant A) and the pruning (Participant B) typically used by the higher-SES and lower-SES, respectively, when employing the heuristic “prior related experience” using the same symptom scenario. Observe that Participant A, a higher-SES person, initiated the Internet search with the terms “vomiting,” “headache,” and “stiff neck.” He also employed the same heuristic, *prior clinical or symptom-related experience*, to decide on the next step of the search. This participant added to the number of symptoms under consideration by methodically reviewing each of the symptoms unique to each type of meningitis. Ultimately, he searched by expanding on the symptoms under consideration to include “fever, headache, stiff neck, nausea, vomiting, and sensitivity to light” in the process of examining a range of causes for the symptoms given to him by the study scenario.

**Table 4:**
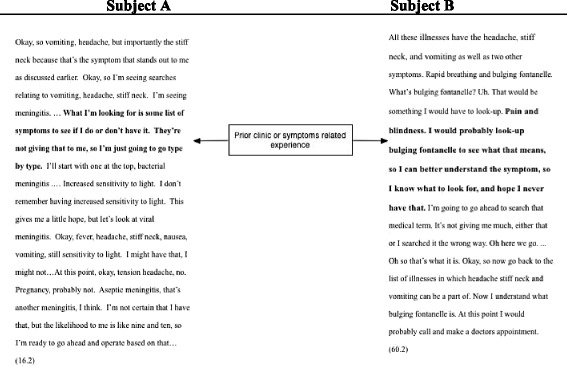
Characterization of the use of the *prior clinical or symptom-related experience* heuristic to narrow or broaden an Internet search

In the parallel example, lower-SES Participant B began his search by considering multiple symptoms, “headache, stiff neck, and vomiting.” When this participant encountered information on the Internet that he did not understand, he considered his *prior clinical or symptom-related experiences* to make sense of the information he found during his Internet search. Lower-SES participants, like Subject B, typically choose to continue investigation of specific symptoms that may appear alarming (“…hope I never have that….”). Upon encountering potentially alarming information, Subject B focused the remainder of his search exclusively on “bulging fontanelle.”

## Discussion

We sought to understand the ways in which individuals interact with Internet health information as a readily available source for healthcare seeking and decision-making. Using two clinical vignettes, we asked individuals to engage in an Internet search, and from these searches, we identify three organizing heuristics that informed our study participants’ health care information seeking: prior clinical or symptom-related experience, credibility of information, and overall story coherence. We observed that searching the Internet for health information to unlikely to influence a decision. This is consistent with previous studies modeling social media information seeking behaviors. These studies found that individuals are drawn to information that is consistent with their own beliefs [15].

Further, we note that participants who were lower-SES (those without a college education and who recruited from sites where social services were offered) were more likely to engage in less complex and more intuitive search strategies that involved a narrowing of their search, rather than expanding information input. Those who were higher-SES (those with a college degree) engaged in a more complex and expanded search process that involved branching rather than pruning information. As a result of their expanded search processes, higher-SES individuals were exposed to additional information as well as a larger number of decision points.

When confronted with a specific set of symptoms, higher-SES participants tended to use search strategies that branch out—the exploration of conditions they expect contribute to the symptoms and systematically exploring offshoots of that condition, such as related conditions or symptoms. Lower-SES participants used heuristics to prune the scope of their Internet search—i.e., heuristics to ignore or remove search topics believed to be superfluous to the condition. When confronted with unfamiliar information, lower-SES participants were more likely to use their search to focus on specific elements of information they did not understand.

Expanded searches require more complex use of organizing heuristics for decision-making. When confronted with informational complexity, lower-SES participants often abandoned the search process or chose to pursue specific symptoms and narrower search paths. This narrowing approach may represent a digression and lead to an inaccurate understanding of “what’s happening.” Despite the fact that study search scenarios were not likely to influence participant assessments about symptom etiology, the decision-making heuristics for directing and processing information were varied and informative of the strategies that might be used more generally by lower-SES and higher-SES individuals to search the Internet for health-related information.

Most recently, studies on decision-making capacity and processes examine the influence of information overload, [16, 17] quality and accuracy of health information, [16] ways in which health information is perceived and interpreted to make sense in the context of individual experiences, [18, 19] and presentation of information [20] on individual decision-making practices. These studies show that more information does not necessarily lead to better health decision making [16, 17]. They do illustrate, however, that individuals will make sense of information by relying on their social experiences and the relative “sense” of these experiences in contextualizing their decision making [18]. Sense making is heavily influenced by the relative coherence in the body of information presented to the individual [20]. Diminished ability to make sense of information, such as low health literacy, is associated with poorer health outcomes and use of health care services [21].

Findings from our study suggest that consumers of health-related information with varying levels of socioeconomic status rely on specific heuristics and search patterns to direct their searches. The influence of credible information and its use in decision-making is connected with education and prior experiences or interactions with healthcare services. We suspect that education and experiences afforded by those with access to resources may expose individuals to additional knowledge and role modeling of assessment strategies necessary to appraise source credibility as well as to create a coherent story. Those lacking such resources may be at a disadvantage when it comes to turning to the Internet to make a health decision. However, effective reliance on credibility cues may set a person up to be appropriately influenced by the health information they find.

There are several limitations to this study and related findings. First, generalizability is limited by convenience sampling from one county in California. Second, participants in our study varied slightly by gender, race, ethnicity, and insurance status from the participants in the county from which they were recruited. Third, a vignette-guided Internet search, driven by symptoms not currently experienced by the participant limits the generalizability of our findings, as it may have artificially influenced study participants’ search efforts and patterns. Conversely, experience with the symptoms also may have influenced the search process.

The Internet is a highly accessible and economical source of health-related information. When presented with illness symptoms, people with varying socioeconomic status approach Internet information searches and the information they encounter differently. We described how individuals with varying socioeconomic status use three heuristics—influence of prior clinical or symptom-related experience, credibility of source, and Internet story coherence – to “fill-in” information and guide their process of seeking health information when confronted with a health decision.

## Conclusion

Especially with regard to populations with lower general levels of literacy and self-efficacy, effective health messaging designed to influence accurate decision-making must design messages so that there is minimal need to employ complex heuristics to “fill-in” information. And when attempting to introduce new health messages to the general public, public health professionals should develop health communications strategies that connect lived experiences with messaging, establish source credibility, and contextualize the health message in a narrative that could be appropriately applied for those to whom the message is directed [22]. Future studies should investigate search processes among individuals with varying degrees of health literacy and self-efficacy to explore how these factors inform the processes involved in searching and processing health-related information.

## Abbreviations

CDC, Centers for Disease Control; SES, socio-economic status
